# A simplified fluid-sensitive MRI protocol for the hands to detect inflammation without contrast administration: a large study of symptom-free subjects from the general population as a reference for normality

**DOI:** 10.1007/s00256-024-04843-9

**Published:** 2024-12-09

**Authors:** Anna M. P. Boeren, Dennis A. Ton, Elise van Mulligen, Bianca Boxma-de Klerk, Pascal H. P. de Jong, Edwin H. G. Oei, Monique Reijnierse, Annette H. M. van der Helm-van Mil

**Affiliations:** 1 Department of Rheumatology, Erasmus Medical Centre, Rotterdam, The Netherlands; 2 Department of Rheumatology, Leiden University Medical Centre, Leiden, The Netherlands; 3 Department of Clinical Epidemiology, Leiden University Medical Centre, Rotterdam, The Netherlands; 4 Department of Radiology & Nuclear Medicine, Erasmus Medical Centre, Rotterdam, The Netherlands; 5 Department of Radiology, Leiden University Medical Centre, Rotterdam, The Netherlands

**Keywords:** Magnetic resonance imaging, Dixon technique, Clinically suspect arthralgia, RAMRIS, Healthy volunteers

## Abstract

**Objective:**

MRI of the hands is valuable for risk-stratification in patients with arthralgia at-risk for developing rheumatoid arthritis (RA). Contrast-enhanced MRI is considered standard for assessment of RA, but has practical disadvantages. It also shows inflammation-like features in the general population, especially at older age, which should be considered in image interpretation. The modified-Dixon (mDixon) technique is reliable compared to contrast-enhanced sequences. Moreover, this short protocol without contrast-enhancement is patient-friendly. Whether it also shows inflammation-like features in the general population is unknown. We studied this to support accurate use in the clinic.

**Methods:**

Two hundred twenty symptom-free volunteers from different age-categories were recruited from the general population and underwent mDixon MRI of both hands. Two readers independently scored MRIs for synovitis, tenosynovitis, and bone marrow edema (BME) in the metacarpophalangeal-joints (MCP) and wrists according to the RAMRIS. Features were considered present if scored by both readers; frequencies > 5% were considered relevant in terms of specificity and determined per age-category (< 40/40- < 60/ ≥ 60-years).

**Results:**

Higher age correlated with higher BME-scores (*p*-value < 0.005), but not with synovitis and tenosynovitis-scores*.* BME (grade 1) occurred in some bones in people aged ≥ 60, 14% had BME in the lunate, 7% in metacarpal-1, and 6% in the trapezium. Synovitis and tenosynovitis did not occur in > 5%, except for grade-1 synovitis in the right distal radio-ulnar-joint in people aged ≥ 60 (11%).

**Conclusion:**

On mDixon MRI, inflammatory features in the hands of the general population are rare. This facilitates image interpretation. To prevent overinterpretation, only several locations should be considered when evaluating people aged ≥ 60-years.

**Supplementary Information:**

The online version contains supplementary material available at 10.1007/s00256-024-04843-9.

## Introduction

In the last decade, patients with arthralgia at-risk for rheumatoid arthritis (RA) are increasingly identified and studied, with the prospects of improving disease outcomes by interventions in at-risk phases, before clinically apparent arthritis and the disease have developed [1, 2]. The pathophysiology of RA-development is described to consist of different phases. First, autoimmune or autoinflammatory responses derail, which can occur up to 10 years before RA-development. Then, joint symptoms occur, and this is generally 6–12 months before RA diagnosis [3]. At this stage, clinically apparent arthritis is still absent but inflammatory symptoms such as joint pain and morning stiffness may occur. The pattern of symptoms at this stage is called Clinically Suspect Arthralgia (CSA) [3, 4]. However, only part of the patients with CSA indeed progress to RA over time. Imaging-detected subclinical joint-inflammation is an important predictor for future RA-development. From the modalities used in practice (ultrasound (US) and MRI), MRI has shown to have the highest accuracy and reproducibility [5]. MRI has therefore become important for risk-stratification [3, 6]. Presence of subclinical joint-inflammation in CSA also indicates a group of arthralgia patients that will benefit from treatment [2, 7]. These recent developments in the field of rheumatology illustrate the upcoming need for MRI in the early detection of joint-inflammation.

So far, contrast-enhanced sequences are mostly used for the detection of inflammation in the hands. The Outcome Measures in Rheumatology (OMERACT) group for the detection of joint-inflammation in RA recommended contrast-enhanced MRI containing a minimum of two orientations of the scanned area (axial and coronal view) and a combination of T1-weighted, and fluid-sensitive TSE sequences with fat-suppression (FS), as well as post-contrast T1-weighted with FS sequences [8, 9]. Despite the high accuracy and reproducibility of this protocol, many rheumatologists do not consider this protocol feasible, due to financial concerns, long scanning times, and related accessibility. This hampers the implementation of MRI in rheumatologic care.

With the aim to make MRI affordable, patient-friendly and feasible for implementation, different MR sequences have been explored which allow a shorter scan time and do not require the use of contrast-agent. For example CHESS- and STIR-based sequences, among these is the Dixon sequence [10–16]. A “modification” of the original Dixon technique (mDixon) allows the acquisition of high-resolution high-contrast water and/or fat images in a very time efficient way [17]. It results in one acquisition of approximately 5 min and provides reconstruction of fat-only, in- phase, out-of-phase, and water-only images. It is less susceptible to artefacts secondary to field inhomogeneity compared to other frequent used FS sequences and independent of field strength [18, 19]. This latter advantage can facilitate the reproducibility between various MR systems and institutions. Moreover, in earlier research, we showed a high reliability between mDixon and regular recommended gadolinium-enhanced TSE fat-saturated MR sequences [10].

However, it is unknown what mDixon MRI of the hands in the general population reveals. This is relevant, since gadolinium-enhanced TSE fat-saturated MR sequences showed inflammation-like features in symptom-free persons of the general population, especially low-grade synovitis and bone marrow edema (BME) were present at older age. These variants of normality should be considered in image-interpretation to prevent overinterpretation [20, 21]. Studies have shown that incorporation of these variants increase the diagnostic accuracy of contrast-enhanced MRI [21]. Whether this would also be required for mDixon MRI is unknown. Differentiating true subclinical inflammation from “normal variations” is especially relevant in the setting of CSA, where inflammatory lesions are evolving and mostly subtle. We aimed to determine the normal variations obtained with mDixon MRI and therefore we studied inflammatory-like features in symptom-free volunteers from the general population in different age-categories.

## Methods

### Participants

Between September 2021 and September 2023, symptom-free volunteers underwent mDixon MRI of both hands in the Leiden University Medical Centre (LUMC, Leiden) and the Erasmus MC (EMC, Rotterdam) in the Netherlands. Volunteers were recruited via advertisements in local newspapers, on Facebook and on local webpages. Inclusion criteria were age 18 years and older, no history of RA or other rheumatic diseases, and no joint symptoms for at least 3 months. Logically, volunteers could not have any contra-indications for the MRI scan. Persons who applied were screened for these criteria by telephone and a subsequent visit at the outpatient clinic, to verify the absence of clinically apparent inflammatory arthritis at physical examination of their joints. The presence of Heberden or Bouchard’s nodes in the absence of joint symptoms, was not an exclusion criterium, because in the absence of joint complaints it may be part of “normal aging.”

Because previous studies showed mostly inflammation-like features at higher age, the following age-distribution was aimed to obtain: 18- < 40, 40- < 60, ≥ 60 as 1:2:2.

During the screening visit at the outpatient clinic, information on weight, height, hand dominance (right or left-handedness), smoking history, alcohol consumption, comorbidities, family history, and medical history were collected. This study was approved by the local medical ethics committee (MEC: 2017–028) and informed consent was provided by all participants.

### Magnetic resonance imaging protocol

For MR imaging, two whole-body 3.0 T MR machines were used (SIGNA Premier (GE HealthCare, Waukesha, Wisconsin, USA) and Ingenia Elition X (Phillips, Best, the Netherlands). Both hands of each healthy volunteer were imaged using a mDixon sequence from the wrist to the MCP2-5 joints with a dedicated coil. Protocols were optimized for the separate MR machines. The standardized MRI protocol included a 2D T2-weighted turbo spin-echo (TSE) mDixon sequence with consecutive slices with the patient supine and the hands placed ventrally in pronation on a fixation device (GE). The other MRI included a 3D PD mDixon sequence (Philips), either with the patient lying on one side with two hands in prayers position or supine, imaging each hand separately, dependent on the patients’ limitations. Coronal and axial images were available for both protocols. The total acquisition time was ∼5–6 min for the mDixon sequence for both vendors. Details on MR parameters are presented in Table 1 for both scanners. Details on the full protocol and positioning are presented in Supplementary method 1.

**Table 1:** MR sequence parameters

	Sequence/ orientation	Acquisition time (min)	TR (ms)	TE (ms)	FOV (mm)	Slice thickness/ gap (mm)	Number of slices	ETL◦/TSE	matrix
GE	2D FRFSE Flex coronal	5:20	3534	10.1	28	0.7/0	80	8	400 × 400
Philips	3D PD Dixon coronal	5:21	1300	“shortest”	250	0.7/ −0.35	230	47	356 × 186356 × 186

*TR* repetition time; *TE* echo time; *FOV* field of view; *ETL* echo train length; *TSE* TSE factor

### MRI scoring

In agreement with the OMERACT recommendations, MRIs were scored for three inflammatory features (osteitis, synovitis, tenosynovitis) according to the validated Rheumatoid Arthritis MRI scoring system (RAMRIS) [8, 22, 23]. Lesions of the metacarpal phalangeal (MCP) joints were evaluated on a joint-by-joint basis. The carpal region was evaluated in three sections: radial, middle, and ulnar part. All three inflammatory features, tenosynovitis, synovitis, and BME, were scored in a range of 0–3. Synovitis was scored on a range 0–3 based on the volume of enhancing tissue in the synovial compartment (none, mild, moderate severe). BME was scored 0 (no edema), 1 if 1–33% of the bone was involved, 2 if 33–67% was involved, and 3 if 68–100% was involved. Similar to the methods described by Haavardsholm et al., the tenosynovitis score was based on the thickness of peritendinous effusion of synovial proliferation (normal, < 2 mm, 2–5 mm, > 5 mm (range 0–3)) [22]. Water-only images in the coronal and axial planes were used to score the three inflammatory MRI features.

Two independent readers (DT/AB) scored the MRIs according to the RAMRIS. The two readers were trained using an independent mDixon MRI dataset comprised of early arthritis and CSA MRI scans. The interclass correlation coefficients (ICC, between both readers), determined before the study start, were 0.89 for the total inflammation score, 0.93 for BME, 0.90 for synovitis and 0.87 for tenosynovitis. Intraclass correlation coefficients (ICC, within each reader) were 0.88 and 0.89 for reader 1 and reader 2, respectively.

To prevent observer bias, caused by the readers knowing that the evaluated MRIs were from healthy individuals, the MRIs of the participants were mixed with MRIs of patients with CSA (*n* = 78) and recent onset clinical arthritis (*n* = 30) (see previous studies for descriptions) and were scored blinded to the clinical diagnosis [24, 25]. After scoring and unblinding, the MRI scores from the symptom-free individuals were extracted and analyzed.

### Data analysis

The semi-quantitative scores for synovitis, tenosynovitis, and bone marrow edema scores were summed as the total-inflammation score according to the RAMRIS [8, 22]. The mean score of both readers was used for the analysis. The presence of inflammation was studied and defined as present when scored by both readers at the same joint/bone/tendon sheath. This conservative method allowed to report on findings that were unequivocally present.

Total inflammation scores were studied in relation to age. Correlations with inflammation scores were studied with the Pearsons’s correlation coefficients. Frequencies of inflammatory features were determined per location, per hand (left and right separately) and per age category (defined as: 18- < 40 years, 40- < 60 years, and ≥ 60 years). Frequencies were indicated in heat maps and considered relevant when present > 5%. This cut-off is in line with earlier research with contrast-enhanced MRI and relates to a specificity of > 95% for a certain feature and location per age-category [21]. For all analyses, Stata V.18.0 (Texas, USA) was used.

## Results

### Participants

In total, 224 volunteers were screened for participation. Four of them did not undergo MRI and were excluded due to anxiety, possibility of pregnancy or contra-indications (copper spiral and recent dental implant which were not mentioned at screening). One hundred sixty-four participants underwent MRI with the MR machine of GE and 60 participants with MR machine of Philips. Of the 164 MRIs made on the GE scanner, 6 were not scored due to quality issues (mostly due to movement artefacts). Consequently, a total of 220 MRIs were obtained and studied. The age ranged from 22 to 90 years; 54 (24%) volunteers were in the age category 18- < 40 years, 85 (39%) in 40- < 60 years and 81 (37%) volunteers in the age category of ≥ 60–90 years. Baseline characteristics are presented in Table 2.

**Table 2:** Characteristics of healthy volunteers

	Total *n* = 220
Female sex, n (%)	128 (58)
Age, mean (SD)	52 (16)
Age categories	
18- < 40 years 40- < 60 years ≥ 60–90 years	54 (25) 85 (39) 81 (37)
Dominant hand right, n (%)	193 (88)
BMI, mean (SD)	25 (3.6)
Weight, median (IQR)	74 (64–84)
Smoking history, n (%)	
Current smoker Ex-smoker Never smoked	11 (5) 80 (36) 129 (59)
Alcohol use, n (%) Units of alcohol consumed/week, median (IQR)	160 (73) 4 (2–6)
Comorbidity	
Cardiac disease, n (%) Pulmonary disease, n (%) Endocrine, n (%)	24 (11) 9 (4) 8 (4)
Morning stiffness hands ≥ 60 min, n (%)	0
Swollen joint count, n (%)	0

*BMI*, body mass index; *IQR*, interquartile range; *min*, minutes; *SD*, standard deviation

### Total MRI inflammation scores

In the total population, the median total inflammation score was 1 (interquartile range (IQR) 0–2). For BME, synovitis and tenosynovitis the median total scores were 0 (IQR 0–1), 0 (IQR 0–0.5), and 0 (IQR 0–0.5), respectively.

### Correlation with age

**Fig. 1:**
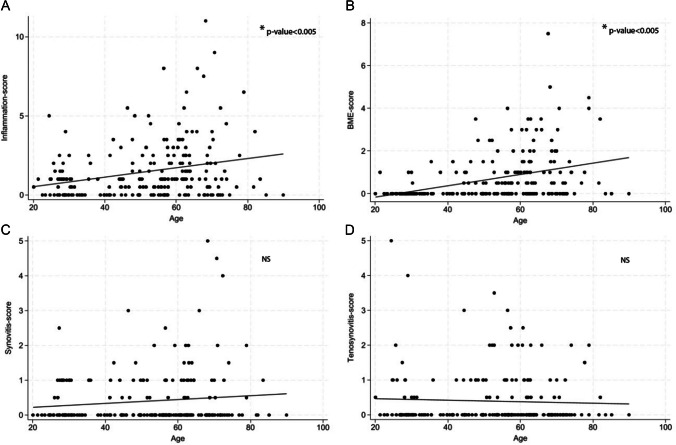
Correlation between age and total inflammation score (**A**), BME (**B**), synovitis (**C**), and tenosynovitis (**D**) on mDixon MRI in symptom-free participants. Legend: correlations between age and inflammatory features detected with Dixon-MRI in 220 symptom-free volunteers. A—correlation between age and the total inflammation score (according to RAMRIS; total score of BME, synovitis, and tenosynovitis): *r* = 0.26, *p-value* < 0.005. B—correlation between age and BME: *r* = 0.37, *p-*value < 0.005. C—correlation between age and synovitis: *r* = 0.11, *p*-value 0.09. D—correlation between age and tenosynovitis: *r* = −0.04, *p*-value: 0.52. BME, bone marrow edema; MRI, magnetic resonance imaging; RAMRIS, rheumatoid arthritis MRI score

The total inflammation score and BME were positively correlated with age. Pearson’s *r* for total inflammation score against age was *r* = 0.26 (*p*-value < 0.005) and for BME Pearson’s *r* was 0.37 (*p*-value < 0.005). Synovitis and tenosynovitis were not correlated with age (Fig. 1). Following this, the prevalence’s of the various inflammatory features were determined per location, and for BME per location and in relation to age.

### Participants without inflammation

Forty-five percent of the participants had a score of 0 and thus no inflammation-like features observed on their MRI scans. Participants without inflammation-like features were observed in all age categories, but decreased with increasing age: 54%, 48%, and 35% of the participants in the age category of 18- < 40, 40- < 60, and ≥ 60–90 years respectively had no inflammatory-like features.

### BME per bone location

**Fig. 2:**
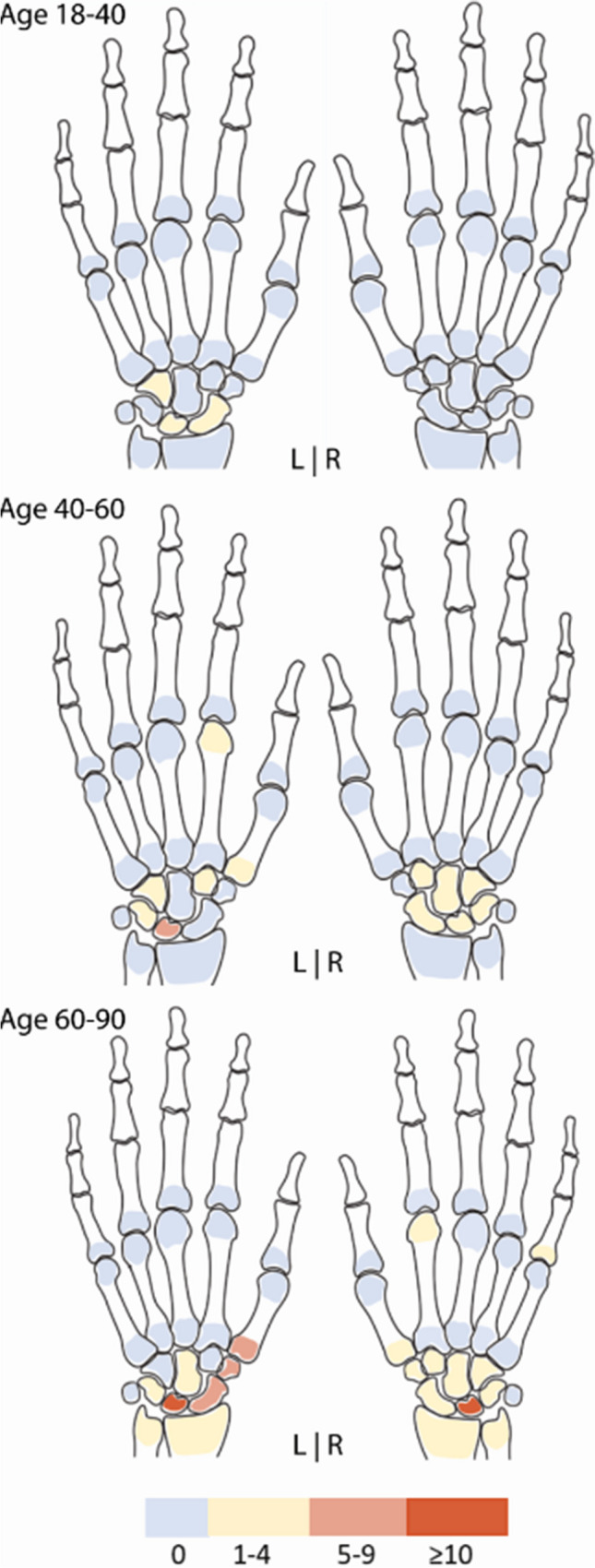
Frequencies of bone marrow edema in symptom-free participants stratified per age category and for the left and right hand. Legend: frequencies (in percentages) of participants with RAMRIS scores of grade-1 BME in the indicated bones (grade 2 was not observed). BME, bone marrow edema; RAMRIS, rheumatoid arthritis magnetic resonance imaging score

Over all age categories, BME occurred infrequently, though the frequency increased with age and BME was mostly present in the participants aged ≥ 60 years (Fig. 2 and Supplementary Table 1). Only grade-1 BME was observed over all age categories. The lunate bone was most effected, especially in people aged ≥ 60, reaching percentages of 14% and 10% in the left and right hand, respectively. Also metacarpal 1 and the trapezium bone were regularly affected in the age category ≥ 60–90 years; for example, BME occurred in 7% in metacarpal 1 and 6% in the trapezium bone of the left hand (Supplementary Table 1).

Within each age category, some bones of the left hand showed slightly more often BME compared to the right hand. If comparing the non-dominant with the dominant hand (based on reported left or right handedness), similar results were found (Supplementary Table 2).

**Fig. 3:**
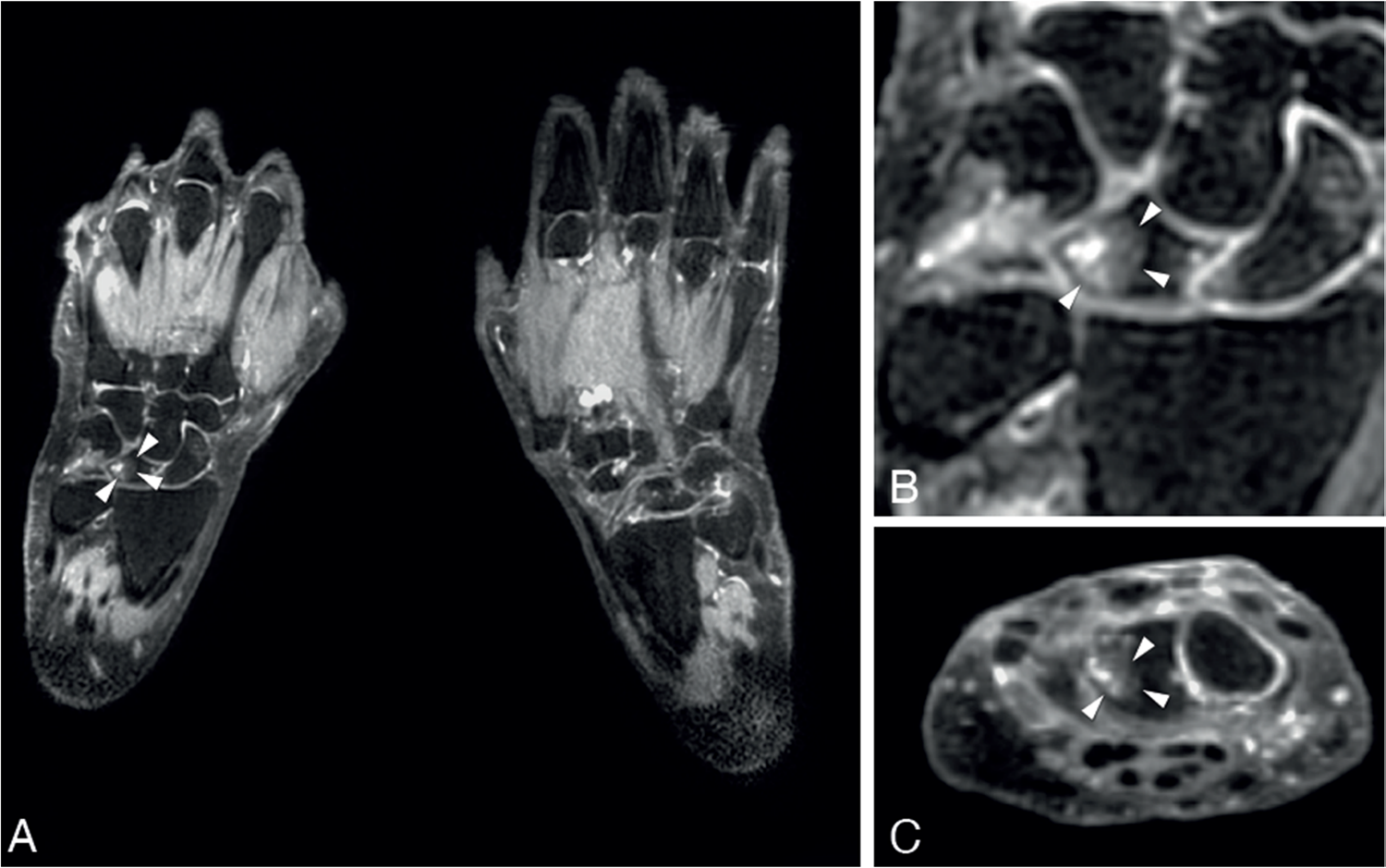
Example of observed bone marrow edema in the lunate bone. Legend: 62-year-old woman of the general population with a subchondral cyst and BME in the lunate bone of the left hand. **A**—coronal slice of both hands. **B**—coronal and **C**—axial slice of the lunate bone of the left hand. BME is indicated with a white arrowheads. BME, bone marrow edema

**Fig. 4:**
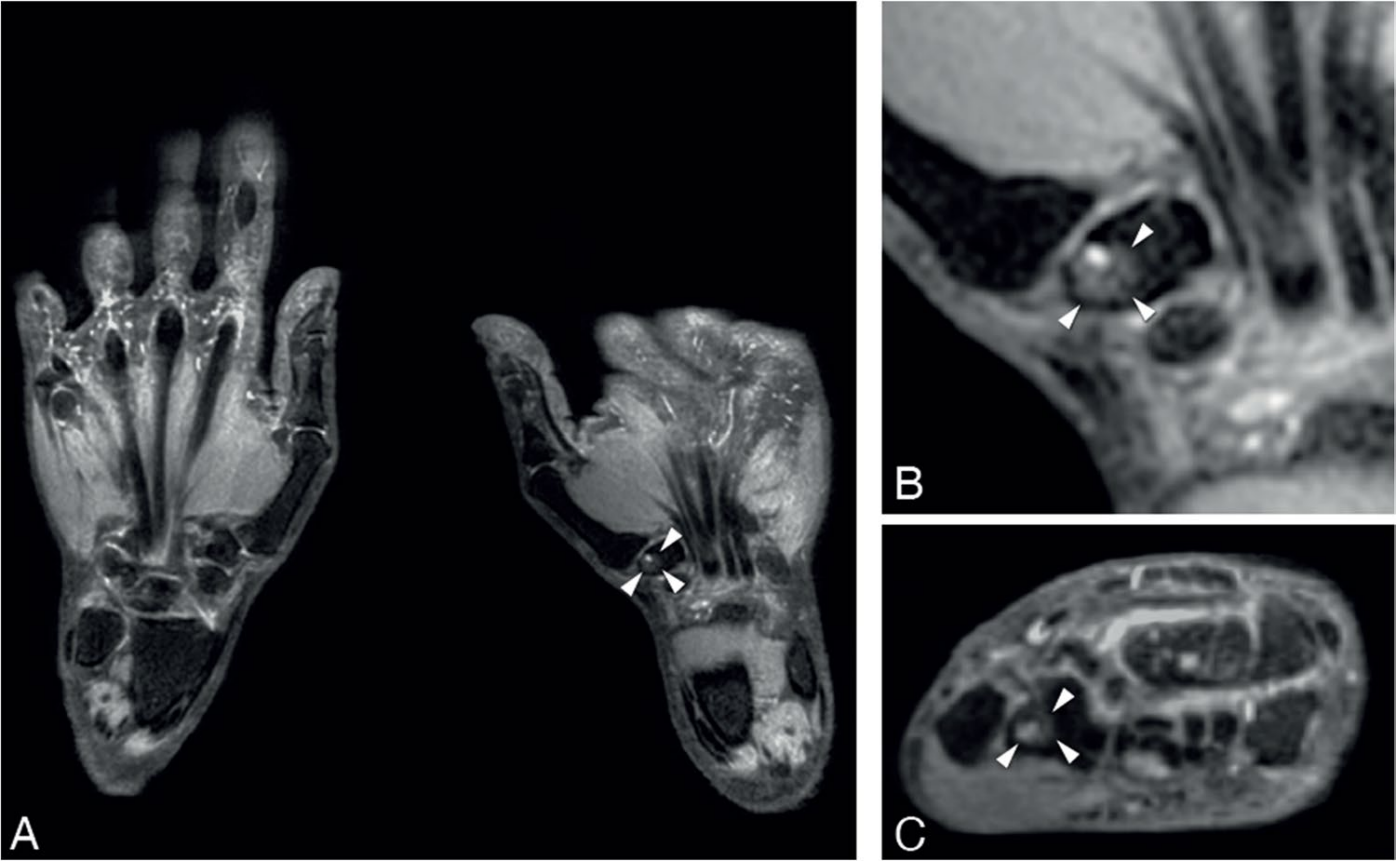
Example of BME in the trapezium bone. Legend: 56-year-old woman of the general population with a subchondral cyst and BME in the trapezium bone of the right hand. **A**—coronal slice of both hands. **B**—coronal and **C**—axial slice at the level of the CMC 1 joint. BME is indicated with a white arrowheads. BME, bone marrow edema

When evaluating locations where BME exceeded the 5% frequency threshold, this involved the lunate for the age category 40- < 60 years and the lunate, scaphoid, metacarpal-1, and trapezium bone in persons aged ≥ 60. An example of BME in the lunate bone is presented in Fig. 3 and an example of BME in the trapezium bone is presented in Fig. 4.

When excluding the people with asymptomatic Heberden and Bouchards nodes (signs at physical examination that in the absence of symptoms may be considered as asymptomatic osteoarthritis or degeneration) from the ≥ 60 age category, the percentages of BME per bone location remained equal (Supplementary Table 3) and the lunate remained most frequently affected (12% and 9% for the left and right hand respectively).

### Synovitis per joint

**Fig. 5:**
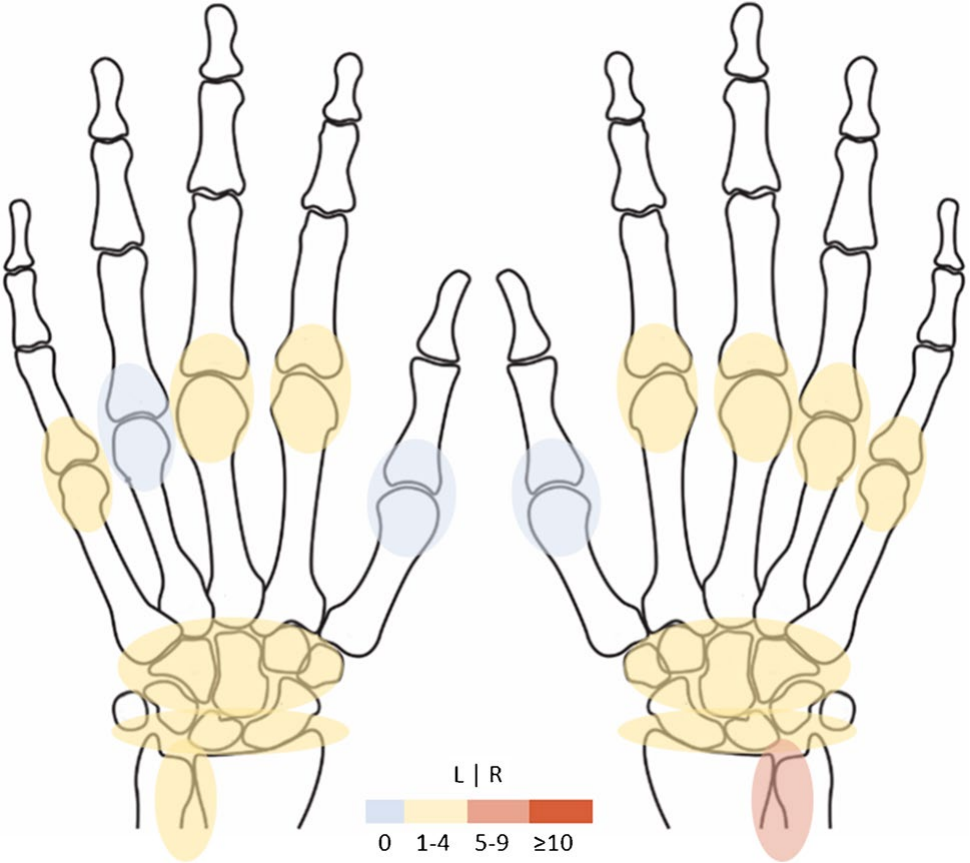
Frequencies of synovitis in symptom-free participants in the left and right hand. Legend: percentages of participants with RAMRIS scores of grade-1 synovitis in the indicated joints (grade 2 was not observed). Only the distal radio-ulnar joint in the dominant hand reached a level of ≥ 5%. RAMRIS, rheumatoid arthritis magnetic resonance imaging score

**Fig. 6:**
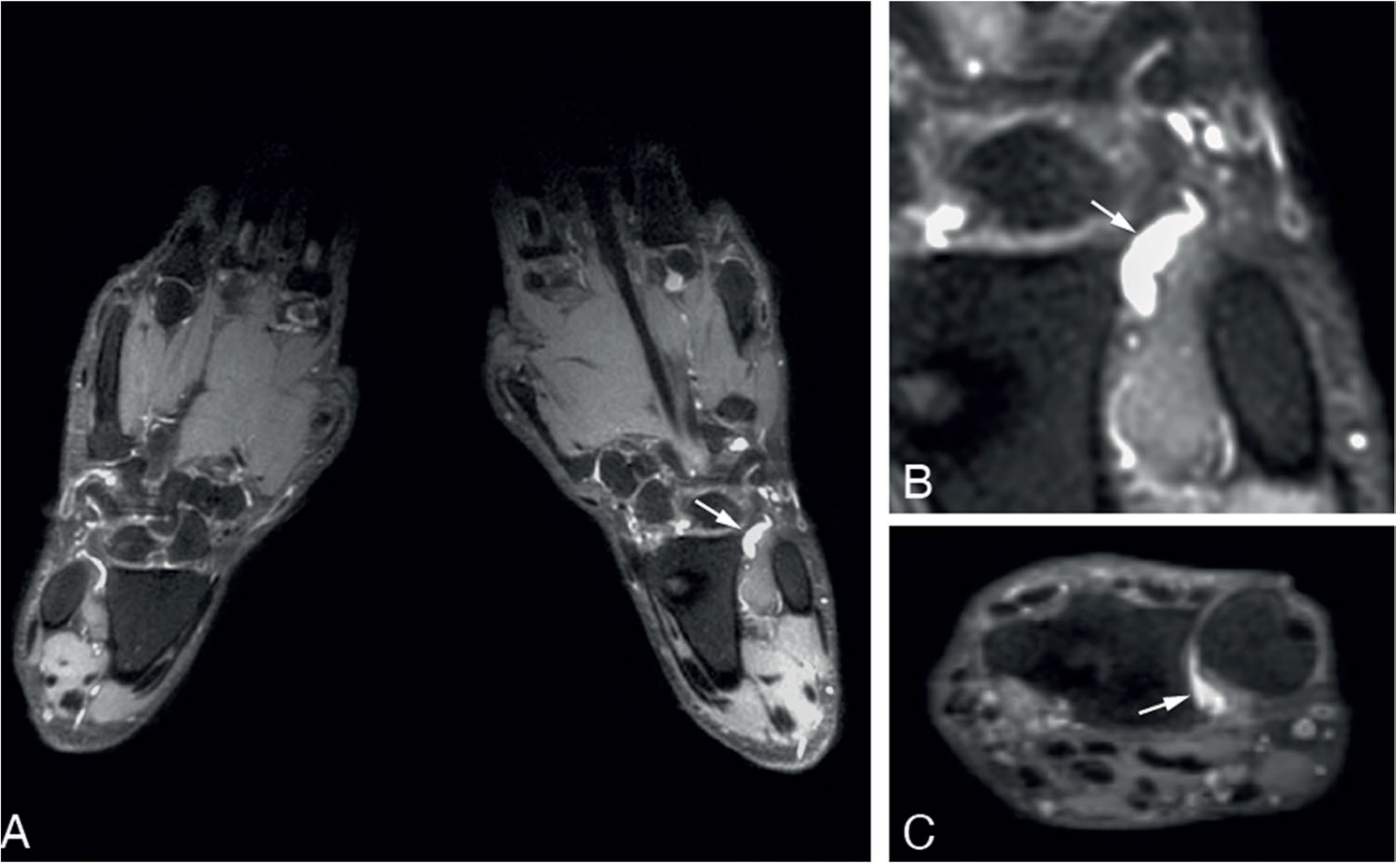
Example synovitis in the DRU-joint. Legend: 50-year-old woman of the general population with bilateral high T2 signal scored as synovitis in the DRU-joint. **A**—coronal slice of both hands. **B**—coronal and **C**—axial slice at the level of the right DRU-joint. Synovitis is indicated with a white arrow. DRU, distal radio-ulnar

Synovitis was infrequent in both the left and the right hand (Fig. 5 and Supplementary Table 4). In the distal radio-ulnar joint (DRU) of the right hand, synovitis grade 1 was observed in 6% of the participants. Although the total synovitis-score was not correlated to age, synovitis at this location occurred more often with increasing age; 11% of the participants aged ≥ 60 years had grade-1 synovitis in the DRU-joint of the right hand and 6% in the left hand (Supplementary Table 4). When stratified by hand dominance, percentages were equal (11% in the dominant hand 6% in the non-dominant hand, Supplementary Table 5). None of the joints evaluated exceeded the 5% frequency threshold, though when considering age, the DRU joint was affected in older symptom-free persons. An example of synovitis in the DRU-joint is presented in Fig. 6.

### Tenosynovitis per tendon sheath

**Fig. 7:**
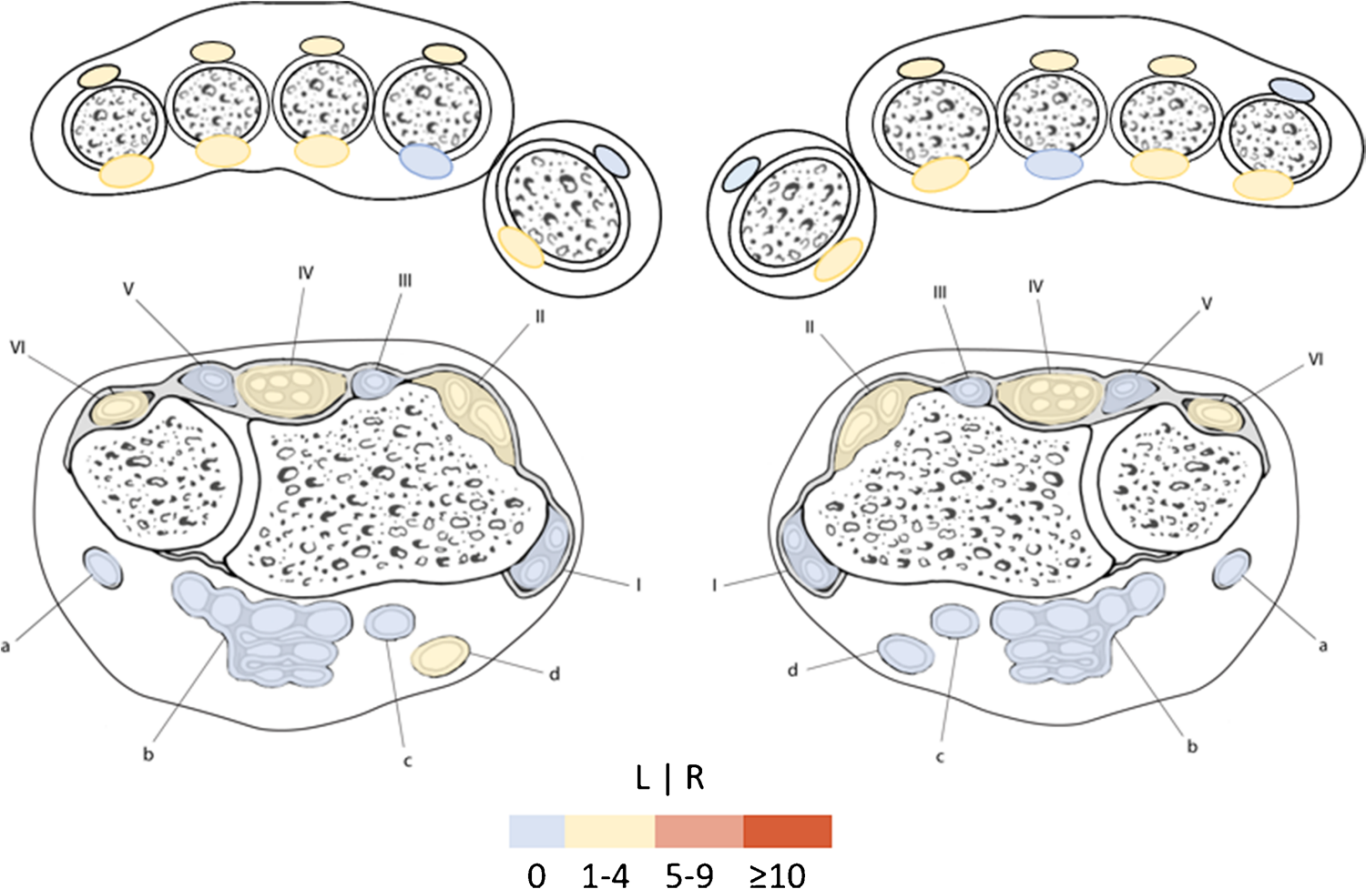
Frequencies of tenosynovitis in symptom-free participants in the left and the right hand. Legend: frequencies (in percentages) of participants with RAMRIS scores of grade-1 or 2 tenosynovitis in the indicated tendon sheaths. In the upper part of the figure, an axial view of the MCP joints is depicted, showing the flexor and the extensor tendons at the level of the MCP joints. In the lower part, an axial view of the wrist is depicted showing extensor tendons (compartment VI, V, IV, III, II, and I) and flexor tendons (a- flexor carpi ulnaris; b-flexor digitorum; c- flexor pollicis longus; d-flexor carpi radialis) of the wrist. Compartment IV was scored two times as grade 2 tenosynovitis (once in the left and once in the right hand (in two different participants.) All other tenosynovitis was scored as grade- 1. RAMRIS, rheumatoid arthritis magnetic resonance imaging score

Tenosynovitis in the MCP- and wrists tendons was infrequent (Fig. 7). In none of the tendon sheaths tenosynovitis occurred in > 5%. Also when considering age categories, tenosynovitis was rare (Supplementary Table 6).

## Discussion

In this cross-sectional study, we showed that synovitis, tenosynovitis, and BME assessed with a mDixon MRI technique rarely occur in the general population, with the exception of BME in several bones including the carpometacarpal (CMC)−1 joint and scaphotrapeziotrapezoidal (STT) joint, the lunate bone and synovitis in the DRU-joint in persons aged ≥ 60 years. With this study, we established a reference of normality for mDixon MRI which can be used when interpretating MRIs with the aim of identifying subclinical joint-inflammation for risk stratification in arthralgia patients who are at-risk for RA development. mDixon MRI is time and patient friendly, making this sequence feasible. The current data showed that relatively few locations show inflammatory-like features in the general population. This means that, during image interpretation, relatively little corrections for normality are needed. This facilitates image interpretation and reinforces that mDixon is suitable for use in clinical practice.

Overall, the scored inflammatory features in the general population detected with mDixon MRI were infrequent. Previous studies with contrast-enhanced MRI showed many more locations with BME and synovitis also in younger people (e.g., those aged 40- < 60) [20]. In a comparison study scanning patients subsequently on 3.0 T MR machine with a short mDixon protocol and 1.5 T MR machine with intravenous contrast, we have shown that mDixon is reliable to detect MRI inflammation. For synovitis, however, it performed moderately [10]. The differences in technology and obtaining images might be an underlying explanation. The theory is that synovial fluid is difficult to differentiate from synovial tissue on mDixon. Vice versa there is a normal amount of fluid in a joint. As long as the high T2 signal is not encircling the joint, synovitis cannot be scored with confidence. Due to the fact that in this study two hands of one person were available and subtle physiological fluid may be equally and symmetrically present in all joints, scoring of the lowest grade of synovitis was not “easily” performed. Consequently, lower frequencies of synovitis might be observed.

BME was especially observed in the oldest age category, with participants ≥ 60 years. The lunate bone was mostly affected, followed by the scaphoid, metacarpal-1 and the trapezium bone. These wrist bones are incorporated in the CMC-1 joint and STT joint and the affected bones might be recognizable for age-related degeneration. In addition, synovitis was most observed in the DRU-joint, possibly also recognizable for degeneration due to degeneration of the triangular fibrocartilage complex (TFCC) with consequent tears and effusion in the DRU-joint [26]. Furthermore, degeneration of the TFCC can also cause BME in the lunate bone [27, 28]. Also in participants aged ≥ 60 years without clinical signs of degeneration, BME remained present with similar percentages per location. However, radiological osteoarthritis can be present without the presence of symptoms and clinical hallmarks and degeneration of the TFCC is usually asymptomatic [29, 30]. Therefore, these hallmarks of degeneration will be present in the general population, without the diagnosis of osteoarthritis disease. This can explain the involved locations of BME and synovitis in our symptom-free population. Importantly, such variations (that may be considered as related to “normal aging”) should not be considered as pathology or presence of subclinical joint-inflammation when evaluating the risk for RA in persons suspected according the rheumatologist.

Earlier research using contrast-enhanced MRI showed that BME and synovitis may occur regularly in asymptomatic populations, also without signs of degeneration and in young adults [27, 31–33]. Therefore, not all observed inflammation-like features will be explained by degeneration, but possibly also by sports, physical strain, chronic trauma, and old trauma [34, 35]. None of the above clinical variables were exclusion criteria since it was important to show these “normalities.” However, in our mDixon data, except from the findings related to the CMC, STT, and DRU joints, other locations did not show frequencies exceeding the 5%. Therefore, such mechanic events seem not to influence MRI interpretation when imaging persons with arthralgia at risk for RA.

In this study, volunteers were included in two centers with MRI scanners of two different MRI vendors. Although the sequences were not completely identical and this could be considered as a limitation, this setting is representative for the clinical settings in which MRI equipment of different vendors are used. Consequently, this adds to the generalizability of this MRI sequence. Interestingly, the frequencies were not largely different between the two MRI vendors (data not shown).

A possible limitation was the observation of local swapping of water and fat signal, which occurred in approximately 13% of the scans (GE only where both hands were scanned at once). However, RAMRIS scoring was not affected by the water-fat swapping as the other image (fat-only) was used in that case [17].

The RAMRIS system was used as it is the only validated method for evaluation of inflammation in hand and foot joints [23]. It was designed for scientific purposes and not for clinical practice. Bone erosions can also be evaluated with the RAMRIS system. Because earlier research in arthralgia at risk for RA showed no predictive value of MRI-detected erosions, and these are therefore not relevant to evaluate in this setting, we here focused on inflammatory features of MRI (BME, synovitis and tenosynovitis) [36].

Image interpretation of contrast-enhanced MRI in patients suspected for having or developing RA is time-consuming because variations of normal occur also at RA-specific locations and these should be considered. For example, synovitis (grade 1) in MCP 2 and 3 occurred in 19% and 17% of people aged ≥ 60 from the normal population, whereas inflammation in MCP 2 and 3 is also frequent in RA [20]. Likewise, tenosynovitis is considered highly specific for RA but at older age it occurred in 12% in the extensor carpi ulnaris tendon in persons aged ≥ 60 years. Hence, contrast-enhanced MRI in rheumatology research used a correction per feature, location, and age category [20]. Although such corrections might not be applied in the regular clinics, it has been shown that the application of corrections increases the diagnostic and prognostic accuracy of MRI [21]. Since inflammation-like features were infrequent in the general population when using mDixon MRI, significant less corrections are needed compared to contrast-enhanced MRI, which facilitates image interpretation.

With the mDixon MR sequence both hands could be imaged in once, opening the opportunity to compare inflammatory features in the right and the left hand and possibly explore differences based on hand dominance. We observed small differences between the left and the right hand. These subtle differences could be due to chance. The clinical value of scanning both or one hand with MRI in patients suspected for RA is a subject for future research.

Now that a reference of normality is established, further research on mDixon MRI should focus on validating its accuracy for the detection of early inflammation and arthritis. Also formal cost-effectiveness studies on mDixon MRI remain to be conducted. These are important steps towards implementation of mDixon MRI in daily (rheumatological) practice.

To conclude, to the best of our knowledge, this is the first study which evaluated mDixon MRI in the hands of symptoms free persons from the general population in order to evaluate the presence of normally occurring features of inflammation. It showed that tenosynovitis was absent and synovitis was rare (except for the DRU at older age). BME occurred in the lunate bone from age of 40 onwards and occurred additionally in the scaphoid, metacarpal-1, and trapezium bones in persons aged ≥ 60. These locations should be kept in mind during image interpretation. Since they are only few this is relatively easily done. mDixon MRI therefore is not only easier to perform compared to contrast- enhanced MRI, but also has advantages in image interpretation.

## Supplementary Information

Below is the link to the electronic supplementary material.

Supplementary file1 (DOCX 598 KB)

## Data Availability

The data underlying this article are available from the corresponding author upon reasonable request.
