# Supplementary material for: A simplified fluid-sensitive MRI protocol for the hands to detect inflammation without contrast administration: a large study of symptom-free subjects from the general population as a reference for normality
Source: Skeletal Radiol. 2024 Dec 9;54(7):1429–39. doi: 10.1007/s00256-024-04843-9 (PMC12078431; doi:10.1007/s00256-024-04843-9)
Supplement: Supplementary file 1 — Supplementary file1 (DOCX 598 KB) [file 256_2024_4843_MOESM1_ESM.docx]

**Supplementary files**

**Supplementary methods 1** MRI protocol and positioning

**Supplementary table 1** Percentages of BME per age category for the left and right hand

**Supplementary table 2** Percentages of BME per age category for the dominant and non-dominant hand

**Supplementary table 3** Percentages of BME in participants aged 60-90 years without Heberden or Bouchard nodes at clinical examination

**Supplementary table 4** Percentages of synovitis per age category for the left and right hand

**Supplementary table 5** Percentages of synovitis per age category for the dominant and non-dominant hand

**Supplementary table 6** Percentages of tenosynovitis per age category for the left and right hand

**Supplementary methods 1** MRI protocol and positioning

*Erasmus MC, Rotterdam – the Netherlands*

MRI was performed on an whole-body3.0T MR system (SIGNA Premier, GE Healthcare, Waukesha, Wisconsin, USA) using coronal interleaved 2D fast spin echo with fast recovery (FRFSE) and proton density weighting (TR/TE=3534/10.1ms) which was implemented with 2-point Dixon processing (Flex, 4 image outputs: water, fat, in-phase and out-of-phase) with an in-plane resolution of 0.7x0.7 mm^2^ (FOV 28 cm), and a slice thickness of 0.7 mm. Full coronal coverage of both hands was achieved using 80 slices in around 5 minutes. Hands were positioned flat side by side over a holder and a GEM Flex Coil 16-S was placed over both hands with an effective width (signal reception) covering from wrist to fingers. Multiplanar reconstructions, including axial reformats, were generated.

Supplementary file methods figure 1 – example of patient positioning


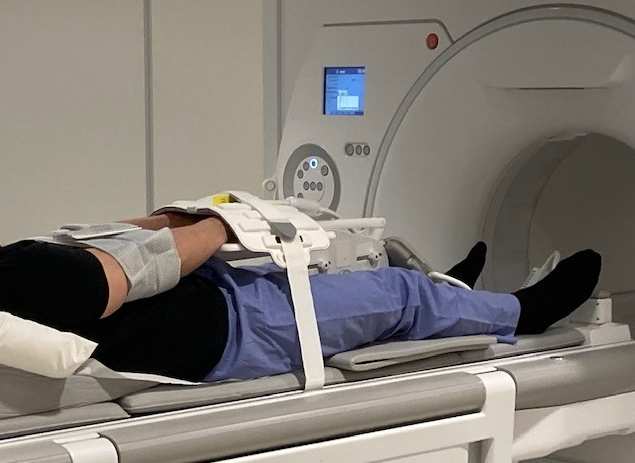


*LUMC, Leiden – the Netherlands*

MRI was performed on an whole bore 3.0T MRI system (Philips, Best, the Netherlands) using a 3D PD Dixon sequence (TR/TE=1300/’shortest”) generating 4 outputs: water, fat, in-phase and out-of-phase with an FOV 250x131 and a slice thickness of 0.7 mm. Full coronal coverage of the hand was achieved using 230 slices in around 5 minutes. Patients were positioned supine with the hand beside the body, fixed with a dedicated coil with cushions. Sequence was repeated for the other side generating one acquisition scan for the left and one acquisition scan for the right hand.

Supplementary file methods figure 2 – example of patient positioning


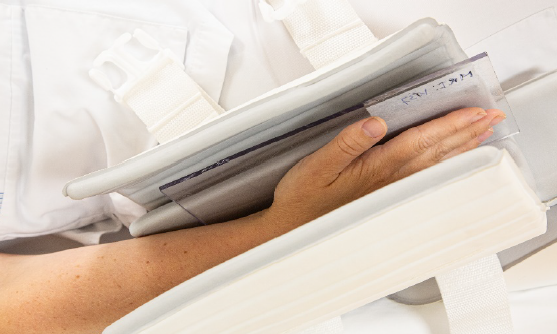


**Supplementary table 1** Percentages of BME per age category for the left and the right hand

|  |  | 18-40 yr  N = 54  Left/Right | 40-60 yr  N = 85  Left/Right | 60-90 yr  N = 81  Left/Right |
| --- | --- | --- | --- | --- |
| MCP BME |  |  |  |  |
|  | MCP 1 – distal | 0/0 | 0/0 | 0/0 |
|  | MCP 1 – proximal | 0/0 | 0/0 | 0/0 |
|  | MCP 2 – distal | 0/0 | 0/0 | 0/0 |
|  | MCP 2 – proximal | 0/0 | 1/0 | 0/1 |
|  | MCP 3 – distal | 0/0 | 0/0 | 0/0 |
|  | MCP 3 – proximal | 0/0 | 0/0 | 0/0 |
|  | MCP 4 – distal | 0/0 | 0/0 | 0/0 |
|  | MCP 4 – proximal | 0/0 | 0/0 | 0/0 |
|  | MCP 5 – distal | 0/0 | 0/0 | 0/1 |
|  | MCP 5- proximal | 0/0 | 0/0 | 0/0 |
| Wrist BME |  |  |  |  |
|  | Metacarpal 1 | 0/0 | 1/0 | 7/2 |
|  | Metacarpal 2 | 0/0 | 0/0 | 0/0 |
|  | Metacarpal 3 | 0/0 | 0/0 | 0/0 |
|  | Metacarpal 4 | 0/0 | 0/0 | 0/0 |
|  | Metacarpal 5 | 0/0 | 0/0 | 0/0 |
|  | Hamate | 1/0 | 0/1 | 0/1 |
|  | Capitate | 0/0 | 0/2 | 2/1 |
|  | Trapezoid | 0/0 | 1/1 | 0/1 |
|  | Trapezium | 0/0 | 0/0 | 6/2 |
|  | Pisiform | 0/0 | 0/0 | 0/0 |
|  | Triquetrum | 0/0 | 2/1 | 4/1 |
|  | Lunate | 1/0 | 7/1 | 14/10 |
|  | Scaphoid | 1/0 | 0/1 | 5/4 |
|  | Distal ulna | 0/0 | 0/1 | 1/1 |
|  | Distal radius | 0/0 | 0/0 | 1/1 |

Legend: percentages of participants with RAMRIS scores of grade 1 BME in the different joints for the right and the left hand. BME grade 2 was not observed. Percentages are presented left/right hand per column. BME, bone marrow edema; MCP, metacarpophalangeal; RAMRIS, rheumatoid arthritis magnetic resonance imaging score;

**Supplementary table 2** Percentages of BME per age category for the dominant and non-dominant hand

|  |  | 18-40 yr  N = 54  dominant/non-dominant | 40-60 yr  N = 85  dominant/non-dominant | 60-90 yr  N = 81  dominant/non-dominant |
| --- | --- | --- | --- | --- |
| MCP BME |  |  |  |  |
|  | MCP 1 – distal | 0/0 | 0/0 | 0/0 |
|  | MCP 1 – proximal | 0/0 | 0/0 | 0/0 |
|  | MCP 2 – distal | 0/0 | 0/0 | 0/0 |
|  | MCP 2 – proximal | 0/0 | 0/1 | 1/0 |
|  | MCP 3 – distal | 0/0 | 0/0 | 0/0 |
|  | MCP 3 – proximal | 0/0 | 0/0 | 0/0 |
|  | MCP 4 – distal | 0/0 | 0/0 | 0/0 |
|  | MCP 4 – proximal | 0/0 | 0/0 | 0/0 |
|  | MCP 5 – distal | 0/0 | 0/0 | 1/0 |
|  | MCP 5- proximal | 0/0 | 0/0 | 0/0 |
| Wrist BME |  |  |  |  |
|  | Metacarpal 1 | 0/0 | 0/1 | 2/7 |
|  | Metacarpal 2 | 0/0 | 0/0 | 0/0 |
|  | Metacarpal 3 | 0/0 | 0/0 | 0/0 |
|  | Metacarpal 4 | 0/0 | 0/0 | 0/0 |
|  | Metacarpal 5 | 0/0 | 0/0 | 0/0 |
|  | Hamate | 0/1 | 1/0 | 1/0 |
|  | Capitate | 0/0 | 2/0 | 2/1 |
|  | Trapezoid | 0/0 | 1/1 | 0/1 |
|  | Trapezium | 0/0 | 0/0 | 2/6 |
|  | Pisiform | 0/0 | 0/0 | 0/0 |
|  | Triquetrum | 0/0 | 1/2 | 1/4 |
|  | Lunate | 0/1 | 1/7 | 11/12 |
|  | Scaphoid | 0/1 | 1/0 | 4/5 |
|  | Distal ulna | 0/0 | 1/0 | 1/1 |
|  | Distal radius | 0/0 | 0/0 | 1/1 |

Legend: percentages of participants with RAMRIS scores of grade 1 BME in the different joints for the dominant and non-dominant hand (based on handedness). BME grade 2 was not observed. Percentages are presented dominant/non-dominant hand per column.

BME, bone marrow edema; MCP, metacarpophalangeal; RAMRIS, rheumatoid arthritis magnetic resonance imaging score; **Supplementary table 3** Percentages of BME in participants aged 60-90 years without Heberden or Bouchard nodes clinical examination

|  |  | 60-90 yr  – excluding individuals with Heberden or bouchards  N = 57  Left/right | 60-90 yr  all individuals  N = 81  Left/right |
| --- | --- | --- | --- |
| MCP BME |  |  |  |
|  | MCP 1 – distal | 0/0 | 0/0 |
|  | MCP 1 – proximal | 0/0 | 0/0 |
|  | MCP 2 – distal | 0/0 | 0/0 |
|  | MCP 2 – proximal | 0/0 | 0/1 |
|  | MCP 3 – distal | 0/0 | 0/0 |
|  | MCP 3 – proximal | 0/0 | 0/0 |
|  | MCP 4 – distal | 0/0 | 0/0 |
|  | MCP 4 – proximal | 0/0 | 0/0 |
|  | MCP 5 – distal | 0/0 | 0/1 |
|  | MCP 5- proximal | 0/0 | 0/0 |
| Wrist BME |  |  |  |
|  | Metacarpal 1 | 7/2 | 7/2 |
|  | Metacarpal 2 | 0/0 | 0/0 |
|  | Metacarpal 3 | 0/0 | 0/0 |
|  | Metacarpal 4 | 0/0 | 0/0 |
|  | Metacarpal 5 | 0/0 | 0/0 |
|  | Hamate | 0/2 | 0/1 |
|  | Capitate | 0/0 | 2/1 |
|  | Trapezoid | 0/0 | 0/1 |
|  | Trapezium | 7/2 | 6/2 |
|  | Pisiform | 0/0 | 0/0 |
|  | Triquetrum | 2/0 | 4/1 |
|  | Lunate | 12/9 | 14/10 |
|  | Scaphoid | 5/4 | 5/4 |
|  | Distal ulna | 2/2 | 1/1 |
|  | Distal radius | 2/2 | 1/1 |

Legend: Heberden or Bouchards nodes are bony enlargement of PIPs and DIPs detected at physical examination that are generally considered signs of degeneration. These nodes do not necessarily cause symptoms. In the setting of symptoms they are considered as features of osteoarthritis. Presented are percentages of participant with RAMRIS scores of grade 1 per joint for the left and the right hand (left/right). BME, bone marrow edema; DIP, distal interphalangeal joints; MCP, metacarpophalangeal; OA, Osteoarthritis; PIP, proximal interphalangeal joints; RAMRIS, rheumatoid arthritis magnetic resonance imaging score;

**Supplementary table 4** Percentages of synovitis per age category for the left and the right hand

|  |  | 18-40 yr  N = 54  Left/Right | 40-60 yr  N = 85  Left/Right | 60-90 yr  N = 81  Left/Right |
| --- | --- | --- | --- | --- |
| MCP-level | MCP1 | 0/0 | 0/0 | 0/0 |
|  | MCP2 | 0/0 | 1/1 | 2/5 |
|  | MCP3 | 0/0 | 0/1 | 1/2 |
|  | MCP4 | 0/4 | 0/1 | 0/1 |
|  | MCP 5 | 0/0 | 1/0 | 1/0 |
| Wrist | Intercarpal | 2/2 | 0/2 | 4/2 |
|  | Radio-carpal | 6/6 | 4/5 | 4/1 |
|  | Radio-ulnar | 0/2 | 2/5 | 6/11 |

Legend: percentages of participants with RAMRIS scores grade 1 synovitis in the different joints for the left and the right hand stratified per age category. Synovitis grade 2 was not observed. Percentages are presented left/right hand per column (according corresponding images).

MCP, metacarpophalangeal; RAMRIS, rheumatoid arthritis magnetic resonance imaging score;

**Supplementary table 5** Percentages of synovitis per age category for the dominant and non-dominant hand

|  |  | 18-40 yr  N = 54  dominant/non-dominant | 40-60 yr  N = 85  dominant/non-dominant | 60-90 yr  N = 81  dominant/non-dominant |
| --- | --- | --- | --- | --- |
| MCP-level | MCP1 | 0/0 | 0/0 | 0/0 |
|  | MCP2 | 0/0 | 2/0 | 5/2 |
|  | MCP3 | 0/0 | 1/0 | 2/1 |
|  | MCP4 | 4/0 | 1/0 | 1/0 |
|  | MCP 5 | 0/0 | 1/0 | 0/1 |
| Wrist | Intercarpal | 2/2 | 1/1 | 2/4 |
|  | Radio-carpal | 6/6 | 5/4 | 2/2 |
|  | Radio-ulnar | 2/0 | 5/2 | 11/6 |

Legend: percentages of participants with RAMRIS scores grade 1 synovitis in the different joints for the dominant and non-dominant hand (based on handedness) stratified per age category. Synovitis grade 2 was not observed. Percentages are presented dominant/non-dominant hand per column.

MCP, metacarpophalangeal; RAMRIS, rheumatoid arthritis magnetic resonance imaging score;

**Supplementary table 6** Percentages of tenosynovitis per age category for the left and the right hand

|  |  | 18-40 yr  N = 54  Left/Right | 40-60 yr  N = 85  Left/Right | 60-90 yr  N = 81  Left/Right |
| --- | --- | --- | --- | --- |
| MCP-level | Ext MCP1 | 0/0 | 0/0 | 0/0 |
|  | Ext MCP2 | 0/0 | 2/2 | 2/0 |
|  | Ext MCP3 | 0/0 | 0/1 | 1/0 |
|  | Ext MCP4 | 0/0 | 0/2 | 1/0 |
|  | Ext MCP5 | 0/0 | 0/0 | 1/0 |
|  | Flex MCP1 | 4/6 | 4/0 | 0/6 |
|  | Flex MCP2 | 0/0 | 0/1 | 0/0 |
|  | Flex MCP3 | 0/0 | 0/0 | 1/0 |
|  | Flex MCP4 | 0/0 | 0/1 | 1/0 |
|  | Flex MCP 5 | 2/4 | 1/4 | 0/0 |
| Wrist | Com VI | 2/2 | 1/1* | 6*/2 |
|  | Com V | 0/0 | 0/0 | 0/0 |
|  | Com IV | 0/1 | 1/2 | 0/1 |
|  | Com III | 0/0 | 0/0 | 0/0 |
|  | Com II | 4/7 | 5/2 | 0/4 |
|  | Com I | 0/0 | 0/0 | 0/0 |
|  | Flex 4 | 0/0 | 1/0 | 4/0 |
|  | Flex 3 | 0/0 | 0/0 | 0/0 |
|  | Flex 2 | 0/0 | 0/0 | 0/0 |
|  | Flex 1 | 0/0 | 0/0 | 0/0 |

Legend: percentages of participants with RAMRIS scores grade 1 tenosynovitis in the different tendons for left and right hand stratified per age category. Percentages are presented left/right hand per column. * indicates once scored as grade 2.

MCP, metacarpophalangeal; RAMRIS, rheumatoid arthritis magnetic resonance imaging score;
